# Catalytic performance of texturally improved Al–Mg mixed oxides derived from emulsion-synthesized hydrotalcites†

**DOI:** 10.1039/c7ra13270k

**Published:** 2018-02-06

**Authors:** Davi D. Petrolini, Alano V. da Silva Neto, Ernesto A. Urquieta-González, Sandra H. Pulcinelli, Celso V. Santilli, Leandro Martins

**Affiliations:** Instituto de Química, UNESP – Univ. Estadual Paulista Rua Prof. Francisco Degni 55 CEP 14800-900 Araraquara SP Brazil leandro@iq.unesp.br +55 16 3322 2308 +55 16 3301 9705; Centro de Pesquisas em Materiais Avançados e Energia – Univ. Federal de São Carlos Rodovia Washington Luis, km 235 CEP 13565-905 São Carlos SP Brazil

## Abstract

Mixed oxides of aluminum and magnesium derived from hydrotalcites were prepared by means of a sol–gel method mediated by an emulsified sol as pore template. The emulsion consisted of ethanol as the continuous phase and *n*-dodecane droplets as the dispersed phase, which was stabilized by the presence of the surfactant Pluronic P123. The use of such an emulsion was essential for obtaining materials with a porous structure that were assessed by mercury intrusion porosimetry and nitrogen physisorption. Additional characterization by NH_3_ and CO_2_ temperature programmed desorption confirmed that despite the enhancement of their textural properties, the number of acid and base sites was reduced in comparison to a reference and conventionally prepared Al–Mg mixed oxide, as a consequence of the depletion of surface hydroxyls during condensation of the precursors around the nonpolar droplets of the emulsion. Catalytic conversion of 2-propanol under conditions of controlled mass and heat diffusion on the texturally improved Al–Mg mixed oxides evidenced the preparation of a more effective catalyst than the poorly porous reference.

## Abstract 2

Mixed oxides of aluminum and magnesium derived from hydrotalcites that were prepared by means of a sol–gel method mediated by an emulsified sol as pore template.

## Graphical abstract

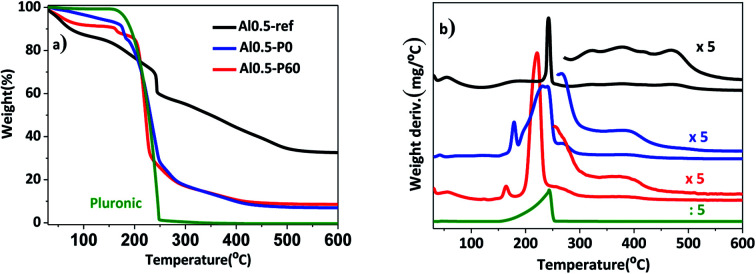

## 1. Introduction

The use of hydrotalcites and their derivatives in catalysis has received widespread attention in industrial processes and in academic studies.^1^ Hydrotalcites are lamellar materials of common formula [Mg_1−*x*_Al_*x*_(OH)_2_]·(CO_3_)_*x*/2_·*m*H_2_O that have a unique and highly tuneable structure with both acidic and basic properties that allow their use as catalysts for the conversion of various organic compounds in the liquid or gas phase.^2–11^ One of the main drawbacks is that the hydrotalcite lamellae may be highly aggregated, exhibiting a relatively low specific surface, which require a modification in order to increase their exposed active surface.

In a catalytic reactor bed, catalysts with enhanced textural properties are very important to lower the pressure drop, to reduce the diffusional resistance of reactants and products and to increase the heat transfer. Excessive or deficient heat in the core of the catalytic bed are challenging because hotspots can be formed near the active sites, leading to premature deactivation or lack of heat, which makes the reactor less productive. Thus, porous catalysts are highly required to improve the overall performance of the reactor.^12^

Porous hydrotalcites can be obtained by the intercalation of bulky molecules during the synthesis that give rise to pillars between the lamellae or by use of the sol–gel method.^13^ These methods increase the porosity of the hydrotalcites and avoid the condensation of layers during their conversion under a heat treatment to mixed oxides. As well known, under high treatment temperatures, a typical hydrotalcite of aluminum and magnesium decomposes to give rise a poorly porous mixed oxide with a periclase structure.^12–15^

During the synthesis of hydrotalcites by the sol–gel method, some surfactants have been added to act as structure-directing agents (SDA), such as F127 copolymer or sodium lauryl sulfate, in order to tailor the hydrotalcite to obtain special particle size, morphology and improved textural properties.^13^ In this context, a promising alternative is the combination of the sol–gel route with a dual soft template technique involving dispersed oil droplets (*i.e.* emulsified “oil in water” system) and triblock copolymers as emulsion stabilizers. Mixed oxides of magnesium and aluminum having macro–mesoporous structures and presenting tuneable amount of macropores and high BET area were already obtained.^16–18^

In the emulsion method, the entrapment of dispersed oil droplets by a continuous phase occurs during the sol–gel transition of the system, induced by a rise in pH or by a change in temperature.^13^ The addition of a surfactant is necessary to intermediate the kinetic stability of the emulsion and to avoid the assemblage of the isolated droplets by placing itself in-between the oil–water interfaces.^19^ After the elimination of the oil droplets and surfactant by firing, a material with a structured pore arrangement is obtained.^13^

Herein is shown the improvements in the catalytic activity of calcined hierarchical hydrotalcites prepared by the sol–gel method from an emulsified *n*-dodecane system and in the presence of Pluronic P123 as emulsion stabilizer. The advantage of this method for tailoring the pore structure of mixed oxides derived from those porous hydrotalcites is demonstrated in the catalytic conversion of 2-propanol into propene and acetone.

## 2. Experimental

### 2.1. Synthesis of the meso–macroporous Al–Mg hydrotalcites and their derived mixed oxides

The synthesis of the hydrotalcites involved the use of aluminum tri-*sec*-butoxide (Al(i-But)_3_) and magnesium nitrate as sources of the hydrotalcite framework. The assembly of the emulsified system used *n*-dodecane, non-ionic triblock copolymer Pluronic P123 (20 EO : 70 PO : 20 EO, MW 5826.4 g mol^−1^) and ethanol as solvent, as described elsewhere.^13^

The as synthesized hydrotalcite gels were calcined for 2 h under air flow, using two temperature plateaus, the first at 220 °C (corresponding to the boiling point of *n*-dodecane) and the second at 500 °C.^13^ During calcination, the hydrotalcite structure collapsed, giving rise to a mixed oxide of aluminum and magnesium. Due to the memory effect of hydrotalcites, just before any characterization or before the catalytic tests, samples were treated at 500 °C under air.^15^

The nomenclature of the samples was according to the mole ratio of Al (Mg_1−*x*_^2+^Al_*x*_^3+^) and the weight percentage of *n*-dodecane, in the form Al*x*-Pwt%. For example, Al0.5-P60 refers to the sample with an Al^3+^ mole ratio of 0.5 and 60 wt% of *n*-dodecane. For the sake of comparison, mixed oxide counterparts synthesized without any SDA were also used in this study, and in this case they are referred as Al0.5-ref. Furthermore, sample Al0.5-P0 refers to the sample prepared in the presence of Pluronic P123 but in the absence of *n*-dodecane.

### 2.2. Characterization of Al–Mg mixed oxides derived from the hydrotalcites

Firstly, the thermal behavior of the as synthesized hydrotalcites was investigated by thermogravimetric analysis, performed from room temperature up to 600 °C under air flow (50 mL min^−1^), by using a SDT600 TA Instruments at a heating rate of 10 °C min^−1^.

The pore size distribution of the Al–Mg mixed oxides was determined by mercury intrusion porosimetry, using an AutoPore III instrument (Micromeritics). Degassing of the samples prior to analysis was performed under vacuum below 0.05 mPa. The pore diameter was calculated by means of the Washburn equation,^20^ using surface tension of 0.489 N m^−1^ and contact angle of 135°. The porous structure of the mixed oxides was analyzed by skeletal (*ρ*_s_) and bulk (*ρ*_b_) density measurements, employing helium (AccuPycc 1330, Micromeritics) and DryFlo® pycnometry (GeoPyc 1360, Micromeritics), respectively. DryFlo® is a quasi-fluid composed of small, rigid spheres having a high degree of flow-ability to accommodate in-between the porous structure of the analysed porous solid. The open porosity (*ε*_P_) was calculated from these density values using the relationship *ε*_P_ = (1 − *ρ*_s_/*ρ*_b_).

X-ray diffraction patterns were collected using a Rigaku Multiflex instrument operated at 30 kV and 15 mA, with Ni-filtered Cu-Kα radiation (*λ* = 1.5418 Å). The X-ray analyses were performed using 2*θ* angles from 3 to 80° and a goniometer step-rate of 0.5° (2*θ*) min^−1^.

Nitrogen adsorption–desorption isotherms were recorded at −195 °C and relative pressure (*P*/*P*_0_) ranging between 0.001 and 0.998, using an ASAP 2010 instrument (Micromeritics). Prior to the measurements, the samples were degassed at 200 °C for 12 h under a vacuum of 0.01 mPa. The specific area was calculated according to the BET equation and the pore size distribution was determined using the BJH method.^21^

XPS measurements were carried out using a commercial spectrometer (UNI-SPECS UHV). The Mg Kα line was used (*hν* = 1253.6 eV) and the analyzer pass energy was set to 10 eV. The inelastic background of the C 1s, O 1s, Al 2p and Mg 2p core-level spectra was subtracted using Shirley's method.^22^ The binding energies of the spectra were corrected using the hydrocarbon component of adventitious carbon fixed at 285.0 eV. The composition of the surface layer was determined from the ratios of the relative peak areas corrected by sensitivity factors for the corresponding elements. The deconvoluted spectral components were obtained using multiple Voigt profiles (70% Gaussian and 30% Lorentzian) without placing constraints. The width at half maximum (FWHM) was varied between 1.5 and 2.2 eV, and the accuracy of the peak positions was ±0.1 eV. The small component at high binding energy tail of the O 1s spectra, attributed to physisorbed carboxyl groups, was subtracted from all envelope spectra.

The strength of the acid sites and the total acidity of the Al–Mg mixed oxides were determined by temperature programmed desorption of ammonia (NH_3_-TPD), using a Micromeritics AutoChem II 2920 apparatus. The samples were preheated to 500 °C under a flow of He during 45 min, and then cooled to 120 °C for the NH_3_ adsorption. A stream containing 10 vol% NH_3_ in He (30 mL min^−1^) was introduced during 30 min, until adsorption equilibrium was achieved. The excess and the physically adsorbed NH_3_ were then purged at 120 °C under a flow of He during 60 min. Finally, NH_3_ was desorbed in a 30 mL min^−1^ flow of He, with heating from 120 to 600 °C at a rate of 15 °C min^−1^. The NH_3_ desorption was monitored online by a thermal conductivity detector.

The strength of the basic sites and the total basicity of the samples were determined by temperature programmed desorption of carbon dioxide (CO_2_-TPD), the samples were pretreated in He (30 mL min^−1^) at 500 °C and hold for 2 h, to remove adsorbed water and carbonate species. Upon cooling to 50 °C in He, the sample was contacted with CO_2_ (30 mL min^−1^), a stream containing CO_2_ pure, for 1 h and flushed with He for 1 h to remove weakly adsorbed CO_2_. The CO_2_ desorption was monitored using a thermal conductivity detector.

### 2.3. Catalytic evaluation

The catalytic activity of the Al–Mg mixed oxides was studied in the conversion of 2-propanol into acetone and propene, employing a microactivity reference system (PID Eng&Tech) operated in continuous flow mode at atmospheric pressure.

Prior to the reaction, 150 mg of the catalyst was thermally treated *in situ* at 500 °C for 1 h, under a 30 mL min^−1^ flow of N_2_. The catalytic measurements were performed either at 270 °C (which was intended to maintain 2-propanol conversion below 10% to exclude the effects of mass or heat diffusion constraints) or at 400 °C (for an operating condition involving high conversion, where mass and heat diffusion might be important). Liquid 2-propanol (99 wt%) was fed into the reactor at a flow rate of 0.1 mL min^−1^, using an HPLC pump (Model 307, Gilson), together with N_2_ (30 mL min^−1^) as carrier gas. Before reaching the catalytic bed, this mixture was completely vaporized and homogenized in a hot box kept at 180 °C. During the reaction, the reactor effluent was analyzed on line using a gas chromatograph (Model 2014, Shimadzu) equipped with a flame ionization detector and a capillary column (Rtx-1, 30 m, 0.32 mm, 1 μm). The 2-propanol conversion (*X*%), the selectivity (*S*%), and the reaction rate (*R*) normalized by the BET area (mol of converted 2-propanol per m^2^ h) were calculated according to eqn (1)–(3), respectively, where *F*_in_ and *F*_out_ are the mole flows (mol h^−1^) of 2-propanol entering and leaving the reactor, respectively, and *F*_product_*i*__ is the mole flow of product P_*i*_.1
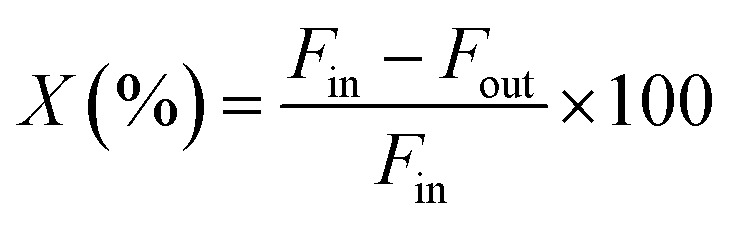
2
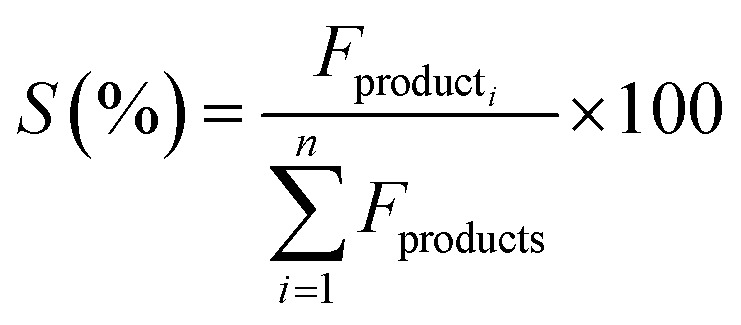
3
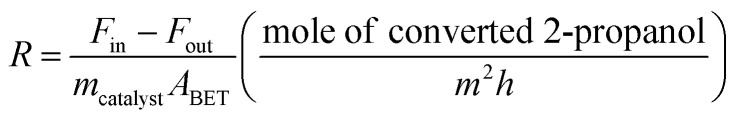


## 3. Results and discussion

### 3.1. Emulsion effect on the creation of the porous mixed oxides

**Fig. 1:**
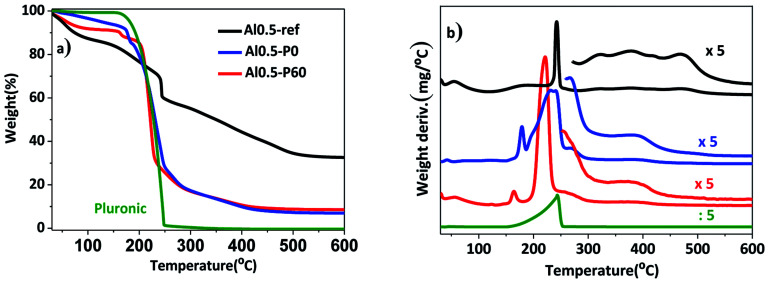
(a) Thermogravimetric curves; (b) weight derivative for the vacuum dried as synthesized hydrotalcites Al0.5-ref, Al0.5-P0 and Al0.5-P60.

In a previous work, the synthesis of hydrotalcites by means of the sol–gel transition in combination with *n*-dodecane as emulsion was effective in producing a material with hierarchical structure of pores.^13^ In this current study, we used the previous hydrotalcites that were heat treated to give rise to the Al–Mg mixed oxides. Fig. 1 shows the thermogravimetric curves and the weight derivatives for the as synthesized and vacuum dried hydrotalcites. The weight loss up to 150–200 °C corresponds to the evaporation of interstitial water.^11^ The drying process was successfully performed because *n*-dodecane, which has a boiling point of 216 °C, was absent. However, in the range 200–250 °C there was a large weight loss for samples Al0.5-P0 and Al0.5-P60 due to the decomposition of the non-volatile Pluronic 123. Finally, between 300 and 490 °C ammonium and/or carbonate cations placed in-between the lamellar structure and water resulting from the dehydroxylation of vicinal hydroxyl groups are lost. Al0.5-P0 and Al0.5-P60 were the samples with the lowest weight loss in this temperature range, because of the lower concentration of surface hydroxyl groups suppressed during the synthesis as a consequence of the condensation of hydrotalcite precursors around the nonpolar droplets of the emulsion and the surfactant.

**Fig. 2:**
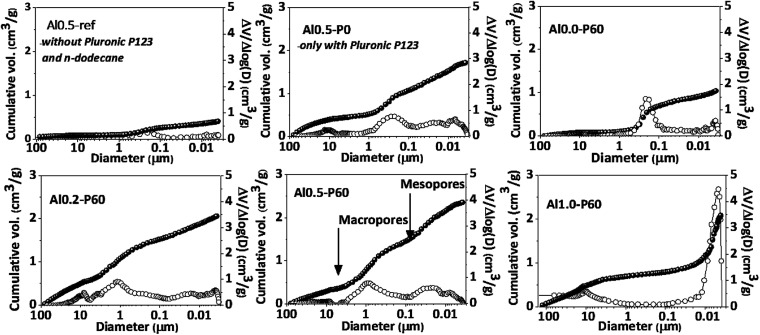
Pore size distributions (open symbols) and cumulative pore volumes (closed symbols), determined by mercury intrusion porosimetry, for the Al–Mg mixed oxides prepared using different aluminum mole fractions.

Fig. 2 and Table 1 collect the results of mercury intrusion porosimetry of the calcined porous Al–Mg mixed oxides in comparison to reference samples, *i.e.* Al0.5-P0 and Al0.5-ref. Pluronic P123 and *n*-dodecane had an additive effect on the porous characteristics of the mixed oxides as revealed by the growth in the BET area and pore volume. The Al*x*-P60 samples had an extensive contribution of mercury intrusion in the range corresponding to the meso and macropores. The meso- and macropore sizes of the porous mixed oxides were in the range of 0.01 and 1 μm. The volume of macropores diminished with aluminum incorporation from 2.0 to 0.5 cm^3^ g^−1^, while mesopore volume increased from 0.8 to 1.6 cm^3^ g^−1^.

**Table 1:** BET area, pore volume, porosity and surface analyses by XPS of the Al–Mg mixed oxides

Synthesis conditions	Sample	BET area^a^ (m^2^ g^−1^)	Porosity *ε*_P_ (%)	Mesopore volume (cm^3^ g^−1^)	Macropore volume (cm^3^ g^−1^)	Total volume (cm^3^ g^−1^)	XPS Al/(Al + Mg)
Without emulsion	Al0.5-ref	166 (7)	75.3	0.1	0.3	0.4	0.41
	Al0.5-P0	260 (119)	82.5	0.9	0.8	1.7	0.37
With emulsion	Al0.0-P60	92 (29)	86.8	0.8	2.0	2.8	—
	Al0.2-P60	235 (33)	86.1	0.8	1.3	2.1	—
	Al0.5-P60	260 (176)	83.0	1.1	1.2	2.3	0.46
	Al1.0-P60	468 (468)	76.2	1.6	0.5	2.1	—

a Refer to samples calcined at 500 °C and measured immediately; values in brackets refer to the corresponding hydrotalcite samples.

Pluronic P123 is a common non-ionic triblock polymer used in the synthesis of several mesoporous materials that consists of hydrophilic polyethylene oxide chains and hydrophobic polypropylene oxide chains.^15–17,21–26^ They are very useful in the synthesis of mesoporous materials because in polar solvents they form micelles that act as template of pores during the condensation of metallic oxides around them, by means of hydrogen bonds with the oxygen atoms of the poloxamer chains. In the emulsion-mediated synthesis, the Pluronic P123 plays an important role by interfering in the kinetic stability of the emulsion droplets by placing between the polar and nonpolar interface. In the case of Al0.5-P0 sample, clearly there is an increase in pore volume compared to Al0.5-ref, however accompanied of a very broad pore volume distribution. This is due to the calcination of the Pluronic P123 micelles trapped in the pores of the as synthesized material that produces gases that break through the condensed solid. The addition of *n*-dodecane in samples Al*x*-P60 gave rise to a porous structure that was dependent on the aluminum fraction. In fact, as described in a previous publication,^13^ the emulsion derived hydrotalcites, which precede the formation of the porous Al–Mg mixed oxides, have a slit-like arrangement of the lamellae in the aluminum fraction between 0.2 and 0.6, but for higher aluminum fraction the hydrotalcite structure collapses leading to a dissimilar arrangement of pores.

**Fig. 3:**
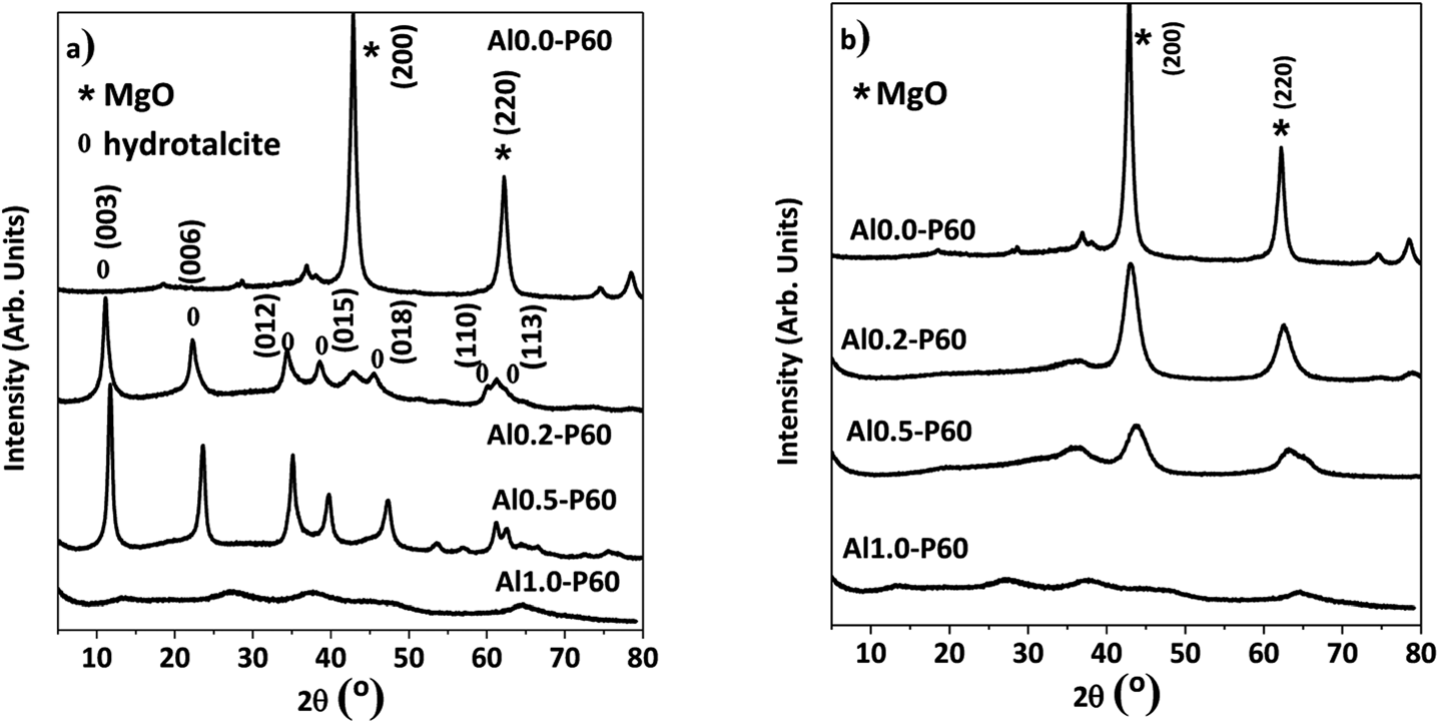
X-ray diffractograms of the Al–Mg mixed oxides with different aluminum mole fractions: (a) reconstructed hydrotalcite samples exposed to room atmosphere; (b) samples calcined at 500 °C and measured immediately.

In the X-ray diffractograms of the mixed oxides shown in Fig. 3b, all samples had a cubic structure derived from MgO phase. Fig. 3a shows the hydrotalcite precursors before the heat treatment. Furthermore, samples consisting of pure alumina (Al1.0-P60) had a structure typical of γ-Al_2_O_3_.

**Fig. 4:**
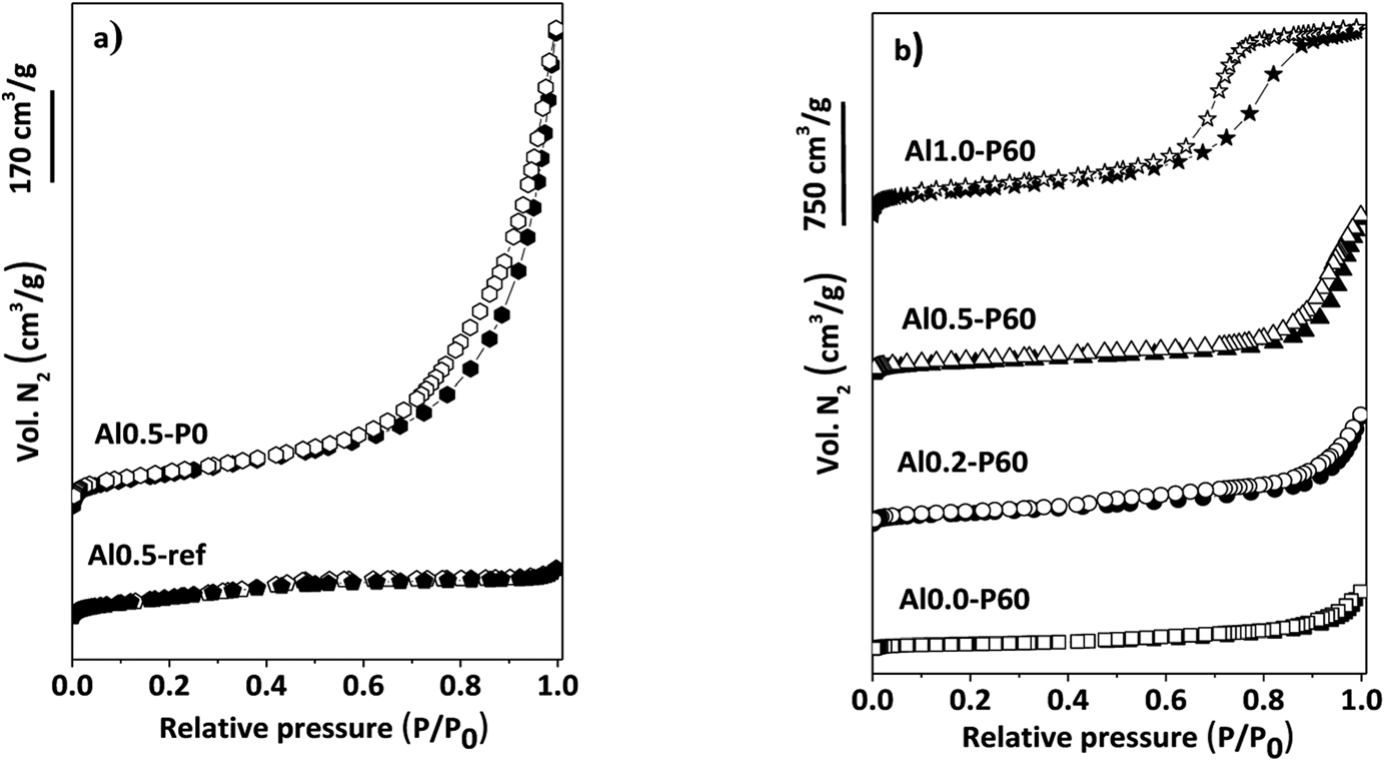
(a) N_2_ adsorption–desorption isotherms of reference samples of Al–Mg mixed oxides; (b) porous Al–Mg mixed oxides with different mole fractions of aluminum.

In the nitrogen physisorption experiments and the BJH pore size distribution curves obtained from the desorption branches of the isotherms (Fig. 4), we can note that adding the SDA components occurred an improvement in the BET area (Table 1) and the generation of a hysteresis loop between the adsorption and desorption isotherms, concerning to the presence of a mesoporous texture in the mixed oxides.^13^ The distribution of mesopores were broad (Fig. 1S†), due to the cross-sections of slit-like pores typical of the parent hydrotalcite (Fig. 2S†).^13^ Macropores are also present, which is evidenced by the lack of a plateau in the *P*/*P*_0_ region near 1.

### 3.2. Acid and basic properties of the Al–Mg mixed oxides

The XPS data of the O 1s core-level of the Al–Mg mixed oxides are shown in Table 2 and Fig. 3S.†Table 1 also demonstrates the surface analyses for Mg 2p and Al 2p. Peak deconvolution and fitting allowed to obtain information regarding the relative amount of oxygen bonded in three different environments, in the form of aluminum or magnesium oxide or hydroxyl groups. The main aspects to be mentioned are the relative position of the binding energy (BE in eV) of the oxygen environment in the magnesium hydroxyl group and its relative abundance. The BE shifts to lower eV values from 531.6 ± 0.1 to 531.3 ± 0.1 as the SDA are added in samples Al0.5-ref, Al0.5-P0 and Al0.5P-60. On the other hand, the O 1s BE signals for Al_2_O_3_ and MgO, were invariant at 530.2 eV. That behavior is explained by the reduction in the density of hydroxyl groups in the surface of the oxides that interact with the nonpolar emulsion during the synthesis,^2,28–32^ as well demonstrated by the thermogravimetric analyses (Fig. 1).

**Table 2:** O 1s XPS distribution of signals of Al–Mg mixed oxides from the profiles shown in Fig. 1S

Synthesis conditions	Sample	Hydroxyl groups		O 1s – Al_2_O_3_ and MgO	
		Pos. (eV)	%	Pos. (eV)	%
Without emulsion	Al0.5-ref	531.9	69.0	530.9	31.0
	Al0.5-P0	531.9	68.8	530.8	31.2
With emulsion	Al0.5-P60	531.7	67.4	530.2	32.6

**Fig. 5:**
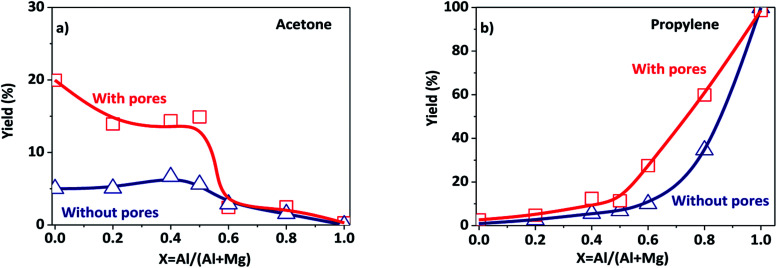
Yields to products resulting from the conversion of 2-propanol at 400 °C (under conditions controlled by the diffusion of mass and heat) on the porous and poorly porous Al–Mg oxides derived from the hydrotalcites: (a) acetone; (b) propylene.

Further evidence of the influence of the SDA on the properties of the Al–Mg mixed oxides was provided by the acid and basic properties determined from temperature programmed desorption (TPD) of NH_3_ and CO_2_ as probe molecules, respectively. Deconvolution using a Gaussian function was used to quantify the overlapping desorption peaks for weak, medium and strong sites (Tables 3 and 1S†). The broad temperature-programmed desorption profiles of chemisorbed NH_3_ (Fig. 4S†) and CO_2_ (Fig. 5S†) suggest that different binding sites were available on the surfaces of the samples. Di Cosimo *et al.*^2^ found that the surface of the Al–Mg mixed oxides was non-uniform and contained sites with varying basic strength. The amount of desorbed NH_3_ was lower for pure MgO (Al0.0-P60), but the strong acid nature increased considerably with higher proportions of aluminum, in accordance to previously reported results.^3,21–25^ The use of the SDA decreased the quantity and strength of acid sites associated with residual hydroxyl groups, varying significantly from 0.78 to 0.51 mmol g^−1^ for Al0.5-ref, Al0.5-P0, and Al0.5-P60. Hydroxyl groups contributed to the strong acidity of the material,^26^ while weak acid sites were associated with Mg^2+^–O^2−^–Al^3+^ cations in the mixed oxide framework.^22,27–29^

**Table 3:** Acid and base sites obtained from NH_3_ and CO_2_ TPD profiles, respectively; 2-propanol conversion rates and acetone and propylene formation rates at 270 °C (for 2-propanol conversion below 10%) on the Al–Mg mixed oxides

Synthesis condition	Sample	Acid sites total NH_3_ (mmol g^−1^)	Base sites total CO_2_ (mmol g^−1^)	2-Propanol conversion rate (mmol h^−1^ m^−2^)	Acetone formation rate (mmol h^−1^ m^−2^)	Propylene formation rate (mmol h^−1^ m^−2^)
Without emulsion	Al0.5-ref	0.78	0.45	0.24 ± 0.01	0.05 ± 0.03	0.19 ± 0.05
	Al0.5-P0	0.45	0.45	0.16 ± 0.04	0.07 ± 0.01	0.09 ± 0.01
With emulsion	Al0.0-P60	0.40	0.47	0.11 ± 0.01	0.09 ± 0.01	0.02 ± 0.01
	Al0.2-P60	0.54	0.49	0.05 ± 0.01	0.04 ± 0.01	0.01 ± 0.01
	Al0.5-P60	0.51	0.43	0.04 ± 0.01	0.02 ± 0.01	0.02 ± 0.01
	Al1.0-P60	0.99	0.39	0.82 ± 0.20	0.01 ± 0.01	0.81 ± 0.06

The acidic-basic characteristics of Al–Mg mixed oxides are antagonistic, since the quantity and strength of acid sites continuously increase with the aluminum fraction, while the basicity decreases. The Al0.5-P60 sample, which had equal amounts of Al and Mg, showed intermediate quantities and strengths of the two types of site, and additionally was the sample that presented the highest porosity. For some catalytic reactions, especially those using supported metals,^30–39^ it is required to use supports with accessibility provided by the network of pores and with minor influence of acidic or basic active sites in order to avoid the formation of by products in unwanted side reactions. Therefore, the Al0.5-P60 sample presents substantial features to be used as catalyst support or catalyst.

### 3.3. Catalytic activity of the Al–Mg mixed oxides

**Scheme 1:**
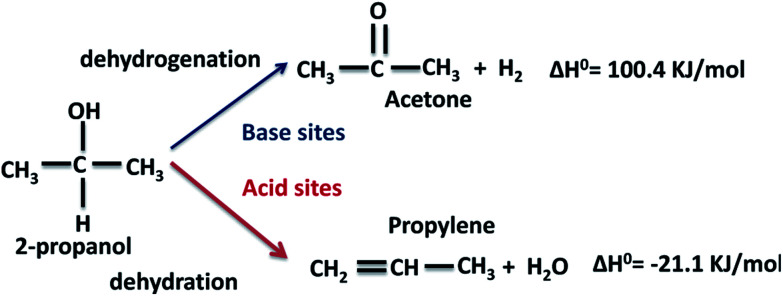
Expected dehydrogenation and dehydration products from the conversion of 2-propanol on a catalyst with simultaneous acid and base sites.

The conversion of 2-propanol on the Al–Mg mixed oxides gives two possible products, depending on the nature of the catalytic sites (Scheme 1). Acetone is formed on base sites in an endothermic dehydrogenation step with Δ*H*^0^ = 100.4 kJ mol^−1^.^40^ Propylene is formed on acid sites in an exothermic dehydration step with Δ*H*^0^ = −21.1 kJ mol^−1^.^41^ Insignificant amounts of di-isopropyl ether are formed due to coupling dehydration.

The performance of the catalysts was studied in two situations. The first one was at 270 °C under surface-controlled conditions and without mass and heat diffusion constraints, keeping the conversion of 2-propanol below 10%. The catalytic results provided in Table 3 correspond to the averages and standard deviations of five consecutive injections of the reactor outlet stream during a period of 1 h. No deactivation of the catalysts was observed during this period or even during a longer period of 6 h for catalysts Al0.5-ref, Al0.5-P0 and Al0.5-P60 (Fig. 6S†).

The specific rate of the reaction was dependent on the aluminum content in the catalyst and could be divided into two regions. In the Mg-rich region, there was preferential formation of acetone, while propylene was formed in the Al-rich region. At Mg/Al = 0.5, there was a crossover and the Al0.5-P60 catalyst was consequently the least active not only due to the ideal mole fraction of Mg and Al, but also due to the specific emulsion/precursors interface created during the synthesis procedure, which affected the acidic-basic properties of the material. These results were in good agreement with the findings published by Diez *et al.*^33^

The porous Al–Mg mixed oxides were also studied at 400 °C in order to obtain high conversions under conditions close to those employed in catalytic processes (Fig. 5). The increased conversion was associated with very different yields of acetone and propylene, with the porous catalysts showing much superior performance. Although both products were subjected to the same mass diffusion regime, the conversion of 2-propanol to acetone was highly favored, relative to propylene. Therefore, the diffusion of heat might have an additional role in the highly endothermic reaction (100.4 kJ mol^−1^) for acetone formation. On the other hand, propylene formation proceeded according to a slightly exothermic reaction (−21.1 kJ mol^−1^). Acetone formation was clearly favored in the aluminum compositional range of 0.2–0.5 that was used to design the parent hierarchical porous hydrotalcites. These results clearly evidence that the preparation of porous Al–Mg mixed oxides derived from hydrotalcites synthesized by the use of an emulsified sol system can lead to a solid with improved textural properties.

## 4. Conclusions

Porous Al–Mg mixed oxides were prepared with success from hydrotalcites synthesized *via* a sol–gel method with the simultaneous addition of Pluronic P123 and *n*-dodecane (emulsion). The combination of textural characterization techniques such as mercury porosimetry and nitrogen physisorption showed the presence of broadly distributed macro and mesopores. The Al–Mg mixed oxides had long-range ordered structure that was confirmed by X-ray diffraction patterns. The use of an emulsified sol changed the composition of the nonpolar interfaces of the emulsion/hydrotalcite Al–Mg precursor layers, leading to changes in the acidic/basic properties of the obtained Al–Mg mixed oxides, due to a decrease in the quantity of surface hydroxyl groups. The porous Al–Mg mixed oxides were used as catalysts in the conversion of 2-propanol. At a high reaction temperature of 400 °C, the catalytic performance of the hydrotalcite-derived porous catalysts exceeded that of those derived from the hydrotalcite synthesized in the absence of emulsion.

## Conflicts of interest

There are no conflicts to declare.

## Supplementary Material

RA-008-C7RA13270K-s001

## References

[cit1] Anastas P. T., Kirchhoff M. M., Williamson T. C. (2001). Appl. Catal., A.

[cit2] Di Cosimo J. I., Díez V. K., Xu M., Iglesia E., Apesteguía C. R. (1998). J. Catal..

[cit3] Yadav G. D., Kadam A. A. (2013). Chem. Eng. J..

[cit4] Álvarez M. G., Plíšková M., Segarra A. M., Medina F., Figueras F. (2012). Appl. Catal., B.

[cit5] Abelló S., Medina F., Tichit D., Pérez-Ramírez J., Groen J. C., Sueiras J. E., Salagre P., Cesteros Y. (2005). Chem.–Eur. J..

[cit6] Cunha A. F., Wu Y. J., Santos J. C., Rodrigues A. E. (2013). Chem. Eng. Res. Des..

[cit7] Ishihara S., Sahoo P., Deguchi K., Ohki S., Tansho M., Shimizu T., Labuta J., Hill J. P., Ariga K., Watanabe K., Yamauchi Y., Suehara S., Iyi N. (2013). J. Am. Chem. Soc..

[cit8] González A. R., Asencios Y. J. O., Assaf E. M., Assaf J. M. (2013). Appl. Surf. Sci..

[cit9] Han S. J., Bang Y., Kwon H. J., Lee H. C., Hiremath V., Song I. K., Seo J. G. (2014). Chem. Eng. J..

[cit10] Jothiramalingam R., Wang M. K. (2009). Ind. Eng. Chem. Res..

[cit11] Ono Y. and Hattori H., Solid base catalysis, Springer Science & Business Media, 2012, vol. 101

[cit12] Perego C., Millini R. (2013). Chem. Soc. Rev..

[cit13] Petrolini D. D., Pulcinelli S. H., Santilli C. V., Urquieta-González E. A., Martins L. (2017). Microporous Mesoporous Mater..

[cit14] Benito P., Labajos F. M., Rocha J., Rives V. (2006). Microporous Mesoporous Mater..

[cit15] Pørez-ramírez J., Abelló S., Van Der Pers N. M. (2007). Chem.–Eur. J..

[cit16] Zhi P. X., Guo Q. L. (2005). Chem. Mater..

[cit17] Valente J. S., Figueras F., Gravelle M., Kumbhar P., Lopez J., Besse J.-P. (2000). J. Catal..

[cit18] Petrolini D. D., Pulcinelli S. H., Santilli C. V., Martins L. (2014). J. Sol-Gel Sci. Technol..

[cit19] Martins L., Rosa M. A. A., Pulcinelli S. H., Santilli C. V. (2010). Microporous Mesoporous Mater..

[cit20] Washburn E. W. (1921). Proc. Natl. Acad. Sci. U. S. A..

[cit21] Sing K. (2001). Colloids Surf., A.

[cit22] Proctor A., Sherwood P. A. (1982). Anal. Chem..

[cit23] Rosa M. A. A., Santos E. P., Santilli C. V., Pulcinelli S. H. (2008). J. Non-Cryst. Solids.

[cit24] Tokudome Y., Fujita K., Nakanishi K., Miura K., Hirao K. (2007). Chem. Mater..

[cit25] Cassinelli W. H., Martins L., Passos A. R., Pulcinelli S. H., Rochet A., Briois V., Santilli C. V. (2015). ChemCatChem.

[cit26] Martins L., Cardoso D., Hammer P., Garetto T., Pulcinelli S. H., Santilli C. V. (2011). Appl. Catal., A.

[cit27] Alves-Rosa M. A., Martins L., Pulcinelli S. H., Santilli C. V. (2013). Soft Matter.

[cit28] Tokudome Y., Nakanishi K., Kanamori K., Fujita K., Akamatsu H., Hanada T. (2009). J. Colloid Interface Sci..

[cit29] Santos R. M. M., Tronto J., Briois V., Santilli C. V. (2017). J. Mater. Chem. A.

[cit30] Cantrell D. G., Gillie L. J., Lee A. F., Wilson K. (2005). Appl. Catal., A.

[cit31] García-sancho C., Moreno-tost R., Mérida-robles J. M., Santamaría-gonzález J., Jiménez-lópez A., Torres P. M. (2011). Catal. Today.

[cit32] Pérez C. N., Pérez C. A., Henriques C. A., Monteiro J. L. F. (2004). Appl. Catal., A.

[cit33] Diez V. K., Apesteguia C. R., Di Cosimo J. I. (2003). J. Catal..

[cit34] Shen J. Y., Kobe J. M., Chen Y., Dumesic J. A. (1994). Langmuir.

[cit35] Shen J., Tu M., Hu C. (2000). J. Solid State Chem..

[cit36] Cristina C., Silva C. M., Ribeiro N. F. P., Souza M. M. V. M., Aranda D. A. G. (2010). Fuel Process. Technol..

[cit37] Di Cosimo J. I., Apesteguia C. R., Ginés M. J. L., Iglesia E. (2000). J. Catal..

[cit38] Prinetto F., Ghiotti G., Graffin P., Tichit D. (2000). Microporous Mesoporous Mater..

[cit39] Cassinelli W. H., Martins L., Magnani M., Pulcinelli S. H., Briois V., Santilli C. V. (2016). RSC Adv..

[cit40] Saito Y., Kameyama H., Yoshida K. (1987). Int. J. Energy Res..

[cit41] Knözinger H., Köhne R. (1966). J. Catal..

