# Supplementary material for: Catalytic performance of texturally improved Al–Mg mixed oxides derived from emulsion-synthesized hydrotalcites
Source: RSC Adv. 2018 Feb 6;8(11):6039–46. doi: 10.1039/c7ra13270k (PMC9078284; doi:10.1039/c7ra13270k)
Supplement: RA-008-C7RA13270K-s001 [file RA-008-C7RA13270K-s001.pdf]

Electronic Supplementary Material (ESI) for RSC Advances.
This journal is © The Royal Society of Chemistry 2018

SUPPLEMENTARY MATERIAL

Catalytic performance of texturally improved Al-Mg mixed oxides derived from emulsion-synthesized hydrotalcites

Davi D. Petrolini[a], Alano V. da Silva Neto[b], Ernesto A. Urquieta-González[b], Sandra H. Pulcinelli[a], Celso V. Santilli[a], Leandro Martins[a]

[a] Instituto de Química, UNESP - Univ. Estadual Paulista, Rua Prof. Francisco Degni 55, CEP 14800-900, Araraquara, SP, Brazil

[b] Centro de Pesquisas em Materiais Avançados e Energia - Univ. Federal de São Carlos, Rodovia Washington Luis, km 235, CEP 13565-905, São Carlos, SP, Brazil

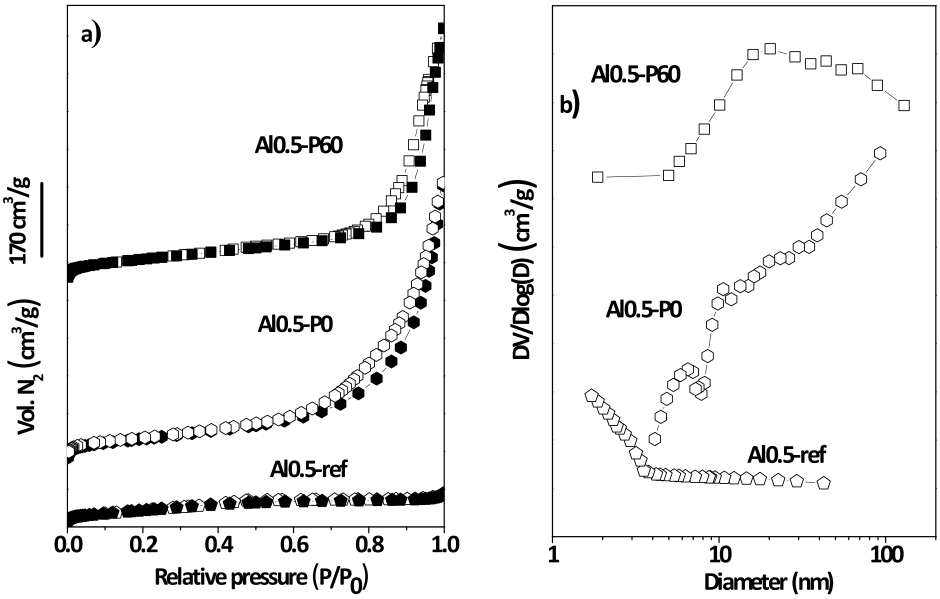

Figure 1S - N_2 physisorption isotherms (a) and the corresponding pore size distributions (b) of samples Al0.5-ref, Al0.5-P0 and Al0.5-P60.

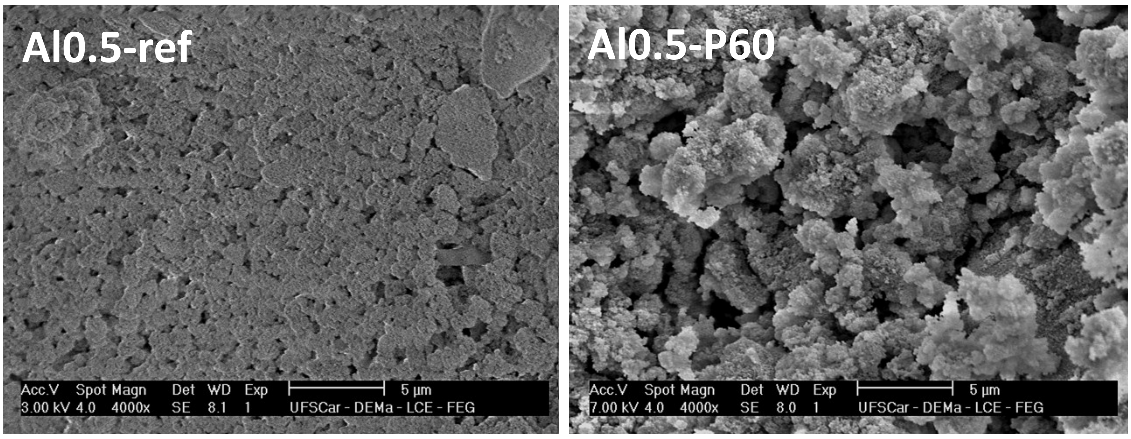

Figure 2S - Scanning electron micrographies of calcined samples Al0.5-ref and Al0.5-P60.

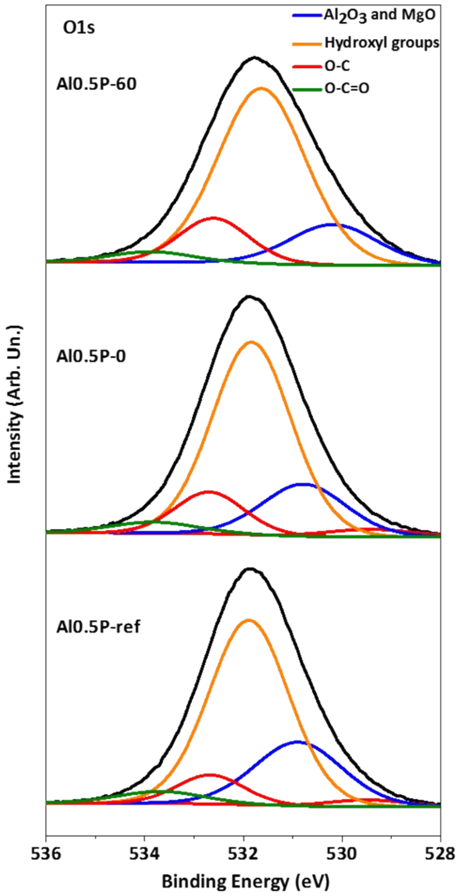

Figure 3S - O1s XPS spectra of the Al-Mg mixed oxides derived from the hierarchical hydrotalcites prepared in the absence or in the presence of 60 wt.% of n-dodecane.

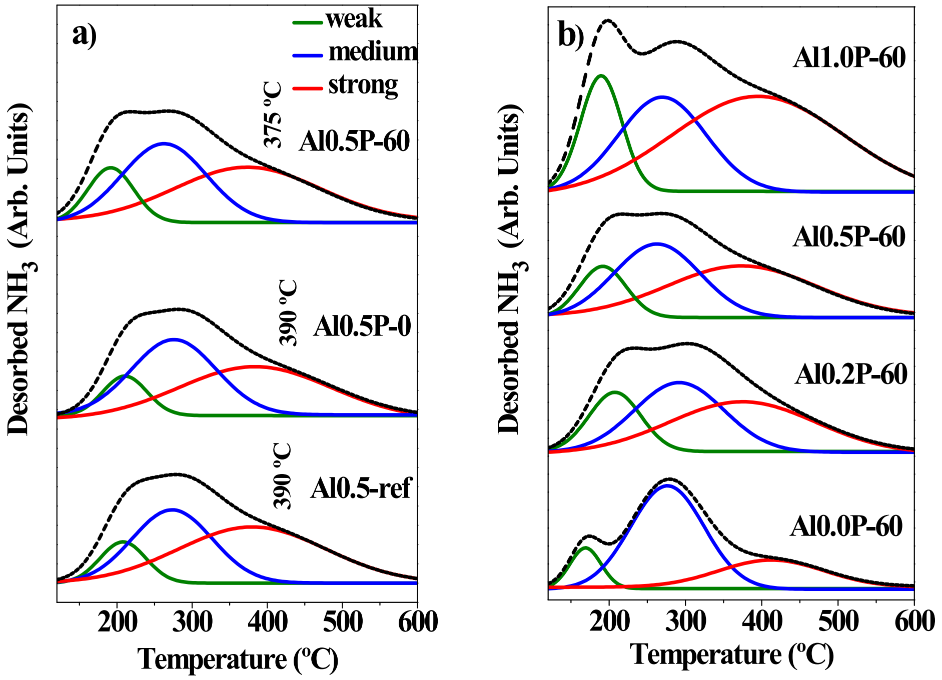

Figure 4S - (a) NH_3-TPD profiles of Al-Mg mixed oxides derived from the hierarchical hydrotalcites prepared in the absence or in the presence of 60 wt.% n-dodecane; **(b)** effect of varying the aluminum fraction.

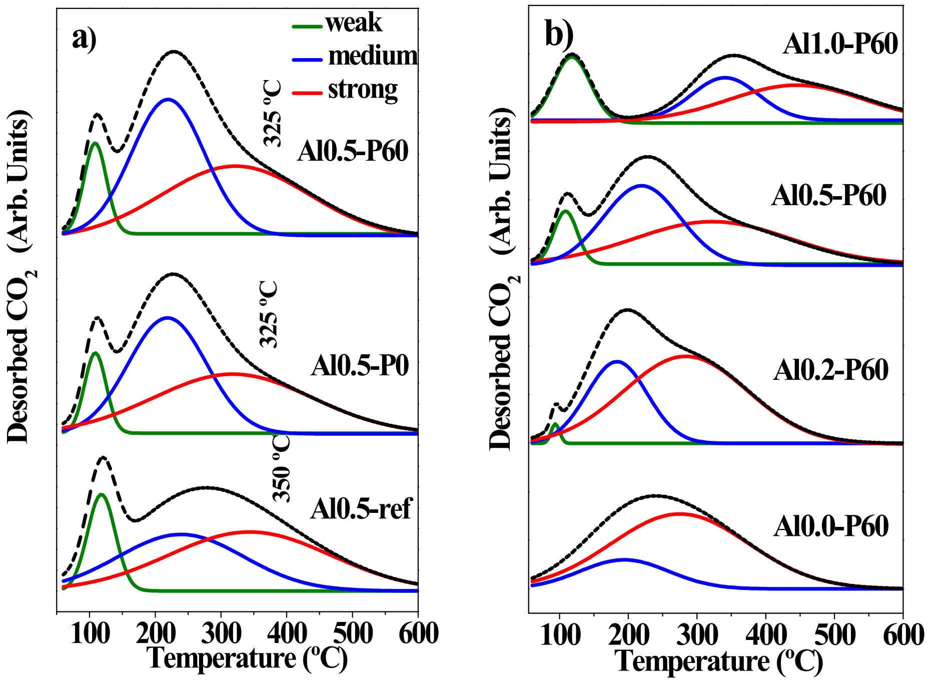

Figure 5S - (a) CO_2-TPD profiles of Mg-Al mixed oxides derived from the hierarchical hydrotalcites prepared in the absence or in the presence of 60 wt.% of n-dodecane; (b) effect of varying the aluminum fraction.

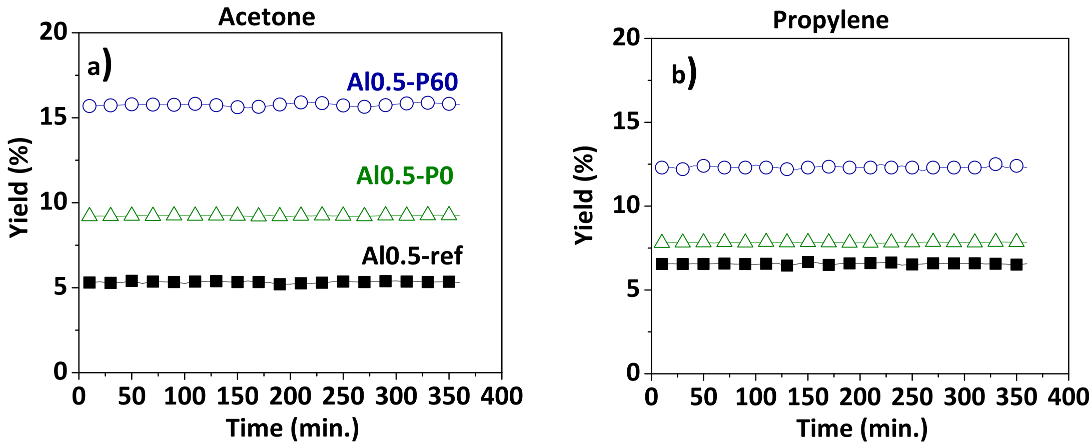

Figure 6S - Yields to products resulting from the conversion of 2-propanol at 400 ºC (under conditions controlled by the diffusion of mass and heat) on the Al0.5-ref, Al0.5-P0 and Al0.5-P60 catalysts: (a) acetone; (b) propylene.

Tabela 1S - Distribution of the strengths of acid and base sites obtained from NH_3 and CO_2 TPD profiles, respectively.

		**Acid sites distribution**				**Base sites distribution**			
**Synthesis conditions**	**Sample**	**Weak (%)**	**Medium (%)**	**Strong (%)**	**Total NH_3 (mmol/g)**	**Weak (%)**	**Medium (%)**	**Strong (%)**	**Total CO_2 (mmol/g)**
Without emulsion	Al0.5-ref	11.6	36.7	51.7	0.78	13.9	36.7	49.5	0.45
	Al0.5-P0	10.8	39.6	49.6	0.45	8.7	44.9	46.4	0.45
With emulsion	Al0.0-P60	10.8	64.9	24.2	0.40	0	20.0	80.0	0.47
	Al0.2-P60	18.2	36.9	44.9	0.54	1.0	31.0	68.0	0.49
	Al0.5-P60	14.6	38.6	46.8	0.51	9.0	47.3	43.7	0.43
	Al1.0-P60	15.7	27.5	56.8	0.99	24.0	48.2	27.8	0.39
